# Comprehensive Transcriptomic Analysis Reveals Defense-Related Genes and Pathways of Rice Plants in Response to Fall Armyworm (*Spodoptera frugiperda*) Infestation

**DOI:** 10.3390/plants13202879

**Published:** 2024-10-15

**Authors:** Xueyan Zhang, Xihao Wang, Tao Wang

**Affiliations:** Ministry of Education Key Laboratory for Ecology of Tropical Islands, Key Laboratory of Tropical Animal and Plant Ecology of Hainan Province, College of Life Sciences, Hainan Normal University, Haikou 571158, China; zhangxueyan324@163.com (X.Z.); wangxihao1999@163.com (X.W.)

**Keywords:** rice plant defense, transcriptome analysis, plant–insect interaction, secondary metabolism, signaling pathways, stress response

## Abstract

Rice (*Oryza sativa* L.) serves as a substitute for bread and is a staple food for half of the world’s population, but it is heavily affected by insect pests. The fall armyworm (*Spodoptera frugiperda*) is a highly destructive pest, threatening rice and other crops in tropical regions. Despite its significance, little is known about the molecular mechanisms underlying rice’s response to fall armyworm infestation. In this study, we used transcriptome analysis to explore the global changes in gene expression in rice leaves during a 1 h and 12 h fall armyworm feeding. The results reveal 2695 and 6264 differentially expressed genes (DEGs) at 1 and 12 h post-infestation, respectively. Gene Ontology (GO) and KEGG enrichment analyses provide insights into biological processes and pathways affected by fall armyworm feeding. Key genes associated with hormone regulation, defense metabolic pathways, and antioxidant and detoxification processes were upregulated, suggesting the involvement of jasmonic acid (JA) signaling, salicylic acid biosynthesis pathways, auxin response, and heat shock proteins in defense during 1 h and 12 h after fall armyworm infestation. Similarly, key genes involved in transcriptional regulation and defense mechanisms reveal the activation of calmodulins, transcription factors (TFs), and genes related to secondary metabolite biosynthesis. Additionally, MYB, WRKY, and ethylene-responsive factors (ERFs) are identified as crucial TF families in rice’s defense response. This study provides a comprehensive understanding of the molecular dynamics in rice responding to fall armyworm infestation, offering valuable insights for developing pest-resistant rice varieties and enhancing global food security. The identified genes and pathways provide an extensive array of genomic resources that can be used for further genetic investigation into rice herbivore resistance. This also suggests that rice plants may have evolved strategies against herbivorous insects. It also lays the groundwork for novel pest-resistance techniques for rice.

## 1. Introduction

Herbivore attacks significantly reduce plant growth and crop productivity. Host plants have evolved numerous defense mechanisms to protect themselves against herbivorous challenges as a result of coevolution and interactions between plants and herbivores [1]. Constitutive defenses for herbivores are the primary expression of physical and chemical defensive traits in the absence of herbivores, whereas induced defenses are triggered only after herbivore attack [2,3]. Rice (*Oryza sativa*) and its insect pests have long been used as model systems to investigate the intricate relationships of plant–insect interactions [4,5]. Moreover, rice (*Oryza sativa* L.) serves as a crucial alternative to wheat for bread production and is the primary staple food for nearly half of the global population. However, rice cultivation faces significant challenges due to the severe impact of insect pests, which cause substantial yield losses and threaten food security in many rice-producing regions [6,7]. Despite the increasing demand, research shows that pests and crop diseases cause 20–40% of global food production losses, with pests directly accountable for or contributing to a substantial percentage of these losses [1]. Among the numerous insect pests of rice, the fall armyworm (FAW), *Spodoptera frugiperda*, is a highly destructive crop pest that is native to the tropical and subtropical regions of the American continent [8]. It poses a significant threat to tropical annual crops due to its ability to feed on various range of host plants, including rice [9,10]. FAW is composed of two genetically distinct strains, the rice (R-strain) and corn (C-strain) strains, which have been extensively studied [11,12]. Understanding the molecular basis of host defense mechanisms against insect attack is crucial for effective insect pest management in crop production.

Plant–insect interaction is an intricate evolutionary system defined by a continuing arms race that forces both organisms to constantly coevolve numerous defense mechanisms and find strategies for their survival [13]. This poses serious problems for global agriculture productivity and food security [14]. This selection process of host plants is intricate and has evolved over millions of years of coevolution between these insects and plants [15]. The act of insects feeding on plants stimulates the generation of various physical and chemical defenses in plants. Plants can activate specific defense mechanisms in response to both mechanical damage and cues from insects [16]. These defenses can be harmful, making the plant toxic, or reducing the digestibility of food for the insect, as well as attracting natural enemies of the insect [17]. Numerous studies have demonstrated that plants’ resistance and defensive responses to insect attacks involve intricate signaling pathways. These pathways are capable of recognizing specific effector molecules present in insect saliva. Upon recognition, these pathways activate robust resistance mechanisms within the host plants, enabling them to effectively defend against insect herbivory [18,19,20]. Activating defense reactions is dependent on immediately and accurately identifying chemical patterns associated with herbivores. Early signaling events are crucial to the basic plant response to herbivory because they initiate subsequent signaling transduction pathways [21,22,23]. Host plant resistance should be considered one of the important ways to reduce crop losses.

Induced defense mechanisms are the results of intricately synchronized sequential changes at the cellular level, changes that activate several signaling pathways [24]. These first processes further activate the calcium flux, the production of reactive oxygen species, membrane potential depolarization, and mitogen-activated protein kinase (MAPK) [23,25]. The plant hormonal signaling network links sensing and early signaling events to wide transcriptional reorganization and defense induction in the dynamic defense process [26,27]. This complex sequence triggers the production of defensive chemicals, proteins, and secondary metabolites, resulting in phenotypic alterations in plants [26,28]. The signaling molecules initiate defense responses by generating secondary metabolites, which can include toxic compounds that contribute to direct insecticidal defense [29,30]. Previous studies reported that secondary metabolites, which include nitrogen- and sulfur-containing chemicals, terpenoids, and phenolics, as well as plant defensive proteins, are inducible defenses against herbivores that are linked to the activation of defense systems in plants [31,32]. Plant hormones that are involved in herbivore-induced defense signaling include auxin, gibberellins, ethylene (ET), jasmonic acid (JA), salicylic acid (SA), cytokinins, and abscisic acid (ABA) [33]. The most studied of these phytohormone pathways are JA and SA [21,23,34]. While the SA route is mostly activated by piercing–sucking herbivores, the JA pathway is normally activated in response to chewing insects and cell-content feeders [35]. In dicotyledonous plants, the JA pathway is widely recognized as the principal and conserved mechanism regulating plant resistance to various kinds of insects [25,26,36]. However, whether and how plant hormones can regulate herbivore-induced defense in rice after fall armyworm infestation is unclear.

However, numerous studies have used a next-generation sequencing approach to examine the interactions between phytophagous insects and their host plants [37,38]. Conducting impartial analyses of the transcriptional profile resulting from RNA sequencing (RNA-Seq) is an effective approach to investigating host plant adaptation mechanisms, and it enables the functional characterization of differentially regulated genes under varying conditions. To profile the transcripts expressed in the rice plants 0 h (control), 1 h, and 12 h after infestation by fall armyworm larvae, we used transcriptome sequence analysis to identify the expression level of genes. Several genes that were either commonly or exclusively expressed, upregulated or downregulated, were identified in rice plants after fall armyworm infestation. Some of these genes are associated with different metabolic processes that produce substances such as SA, JA, ET, and other secondary metabolites. Using two high-throughput sequencing methods, our results provide a comprehensive and preliminary understanding of the changes in the rice plant transcriptome during insect infestation. The candidate genes that have been identified in the current study are important starting points for further investigation into the functions of key genes involved in rice plant–insect interactions.

## 2. Results

### 2.1. Overview of Quality Control and Mapping Statistics of Transcriptome Dataset

**Figure 1:**
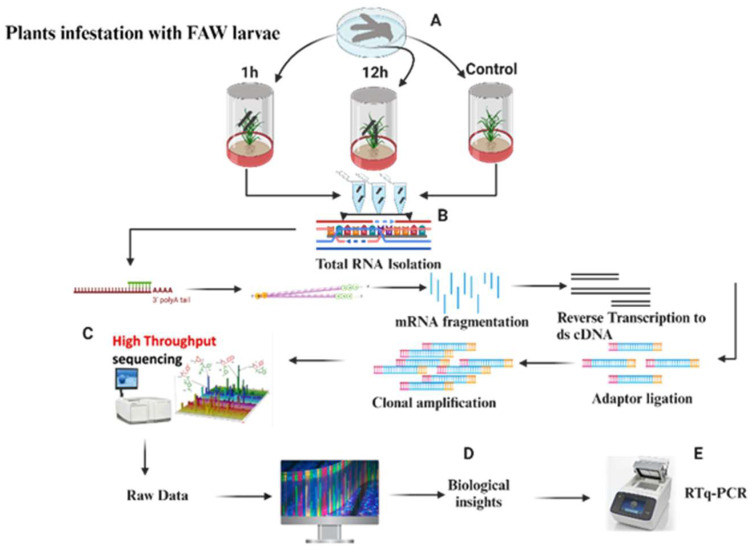
Showing the layout of experimental design: (**A**) infestation of rice plants with FAW larvae for 1 h and 12 h and control; (**B**) larvae were collected from infested host plants for RNA extraction and cDNA synthesis; (**C**) high-throughput sequencing and raw data; (**D**) biological insights; and (**E**) RTq-PCR analysis for the verification of DEGs data obtained from transcriptome analysis.

The FAW larvae caused damage to rice leaves during the 12 h feeding session (Figure 1). To examine the global transcriptome changes resulting from this feeding, an mRNA analysis was carried out on nine libraries, each of which represented unique time intervals (0, 1, and 12 h). Together, these libraries included three biological replicates at every sample interval. For all samples (Control, Rice_1H, and Rice_12H), there was at least 4.9-to-6.8 GB of data with Q20 and Q30 quality scores higher than 97% (Table 1). In total, 53.16–53.71% of the reads were GC-containing, and 44.84–56.19 million of those reads were uniquely mapped to the rice IRGSP–1.0 reference genome. The range of unique mapping rates was from 89.73% to 91.36% (Table 1). Based on our analyses of the gene structure, most mapped reads (92.40–95.70%) were found to be in exons. The uniquely mapped reads were used in further investigations.

### 2.2. Analyses of Differentially Expressed Genes

**Figure 2:**
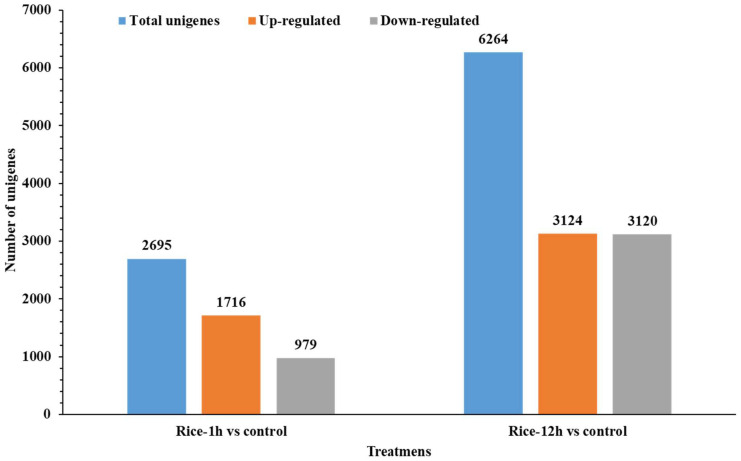
Bar graph showing a total number of up- and downregulated differentially expressed genes (DEGs) in transcriptome analysis of Rice-1h vs. control and Rice-12h vs. control after FAW larvae infestation.

**Figure 3:**
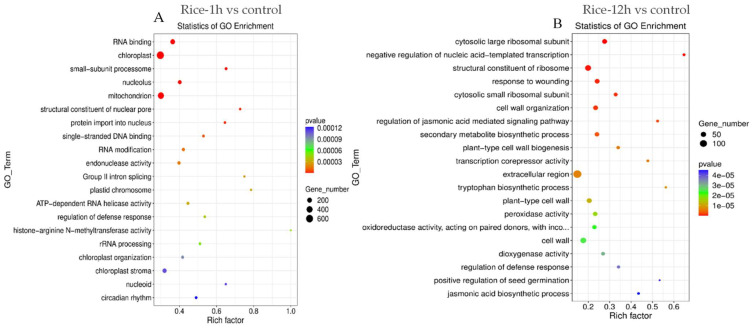
Gene Ontology (GO) classification of transcripts. The number of significantly up- and downregulated unigenes in Rice-1h vs. control (**A**) and Rice-12h vs. control (**B**) after FAW larvae infestation.

DESeq2 software version: 1.45.3 was used for determining the upregulation and downregulation of genes based upon relative Fragments Per Kilobase Million (FPKM) counts (*x*-axis: log2fold change; *y*-axis: −log (*p*-value) Figure 2. A total of 2695 and 6264 differentially expressed genes (DEGs) were regulated in rice leaves at various time points (Rice-1h vs. control and Rice-12h vs. control) after FAW larvae infestation (Figure 3). Among them, there were 1716 upregulated and 979 downregulated genes in Rice-1h vs. control and 3134 upregulated and 3120 downregulated genes in Rice-12h vs. control after FAW larvae infestation (Figure 3).

### 2.3. GO Enrichment Analysis of DEGs Induced by FAW Larval Feeding

**Figure 4:**
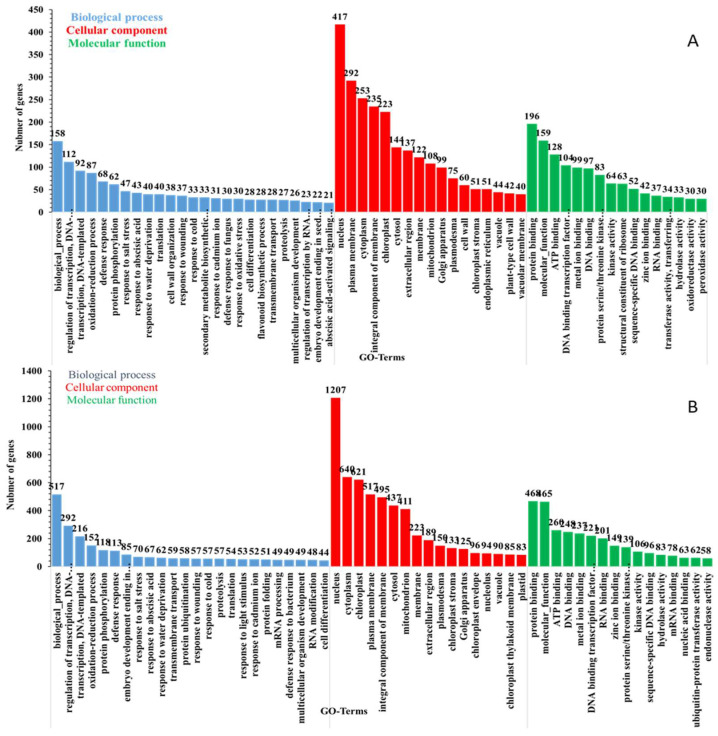
Gene Ontology (GO)-enriched terms of differentially expressed genes (DEGs) and unigenes in Rice-1h vs. control (**A**) and Rice-12h vs. control (**B**) after FAW larvae infestation. The *x*-axis lists the sub-GO terms under categories of biological process, cellular component, and molecular function. The *y*-axis is the number of DEGs involved in each term.

A Gene Ontology (GO) enrichment analysis was conducted to categorize the functional roles of differentially expressed genes (DEGs) in rice plants subjected to feeding by fall armyworm (FAW). This analysis aimed to identify biological processes, molecular functions, and cellular components associated with DEGs. The study specifically examined genes that exhibited a differential expression at both an early time point (1 h after FAW feeding compared to the control) and a late time point (12 h after FAW feeding compared to the control) (Figure 3A,B). The results show the upregulated genes associated with the following at one hour: “cellular response to nucleus”, “regulation of jasmonic acid-mediated signaling pathway”, “response to acid chemical”, and “regulation of defense response”. Upregulated differentially expressed genes (DEGs) showed notable enrichment in several Gene Ontology (GO) biological process, and these comprised procedures like “biological process”, “regulation of transcription DNA”, “defense response”, “protein phosphoration”, and “abscisic acid activated signaling pathways”. Interestingly, “DNA binding transcription factor”, “oxidoreductase activity”, “peroxidase activity”, and “kinase activity” showed notable enrichment in the DEGs at different time points (Figure 4A,B).

### 2.4. KEGG Enrichment Pathways Analysis of DEGs Induced by FAW Larval Feeding

**Figure 5:**
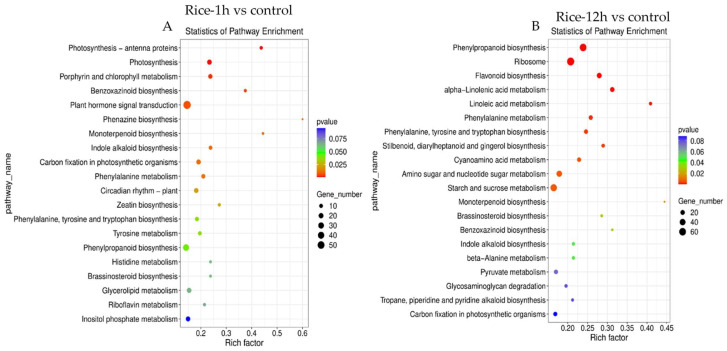
Kyoto Encyclopedia of Genes and Genomes (KEGG) annotation and pathways of in Rice-1h vs. control (**A**) and Rice-12h vs. control (**B**) after FAW larvae infestation.

**Figure 6:**
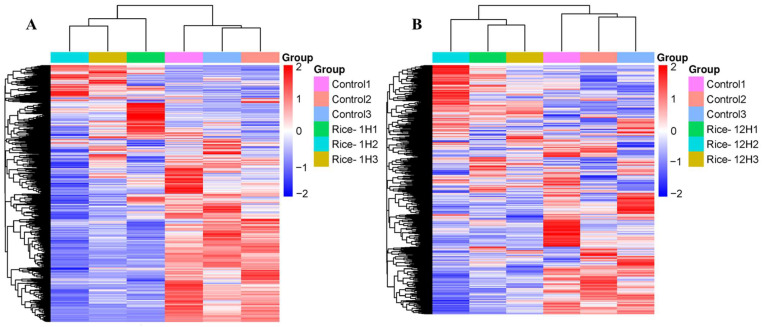
The expression patterns of defensive–responsive genes in in Rice-1h vs. control (**A**) and Rice-12h vs. control (**B**) after FAW larvae infestation. Hierarchical clustering heat map depicting overall results of the FPKM clustering using log2 (FPKM + 1) values. The red and blue squares indicate genes with high or low gene expression levels, respectively.

In this study, the genes that were regulated at one hour and twelve hours both showed a notable enrichment in plant hormone signal transduction, according to the KEGG enrichment analysis. For the Rice-1h vs. control mark, the regulation of genes displayed enrichment in terms of the regulation of defense response (Figure 5A,B), whereas the regulated genes were significantly enriched in pathways linked to the “response to wounding”, regulation of defense response and secondary metabolite biosynthetic process, and regulation of jasmonic acid-mediated signaling pathways in Rice-12h vs. control after FAW larvae infestation (Figure 5A,B). The overall results of the FPKM cluster analysis (Figure 6A,B) of differentially expressed genes were clustered using their log2 (FPKM + 1) value in the form of a heat map (Figure 6A,B). The clustering of genes with similar expression levels was carried out to better understand gene expression in relation to their biological relevance.

### 2.5. Identification of Important Genes Based on Functional Classification and GO Enrichment Analysis of DEGs in Response to FAW Larvae Feeding

Plant responses to insect herbivores depend heavily on early signaling processes, such as the proteins that control the expression of target genes, such as transcription factors (TFs) and transcriptional regulators (TRs). The activation of these signals in response to stress mediated by insects can provide important information about the molecular mechanisms that underlie plant responses to stress caused by insect herbivory. In our study, auxin-responsive protein IAA2 and LRR receptor-like serine/threonine-protein kinase in Rice-1h vs. control and Rice-12h vs. control, were among the differentially expressed genes (DEGs) (Supplementary Table S1). Notably, the families with the highest number of linked TFs or TRs included ethylene-responsive transcription factor, anthocyanidin reductase, zinc finger A20 and AN1 domain-containing stress-associated protein, basic helix–loop–helix (bHLH) genes, and WRKY genes in Rice-1h vs. control and Rice-12h vs. control (Supplementary Table S2).

### 2.6. Identification of Genes Related to Secondary Metabolism Transcriptional Regulation, Hormone Regulation, and Antioxidant and Detoxification Processes

**Figure 7:**
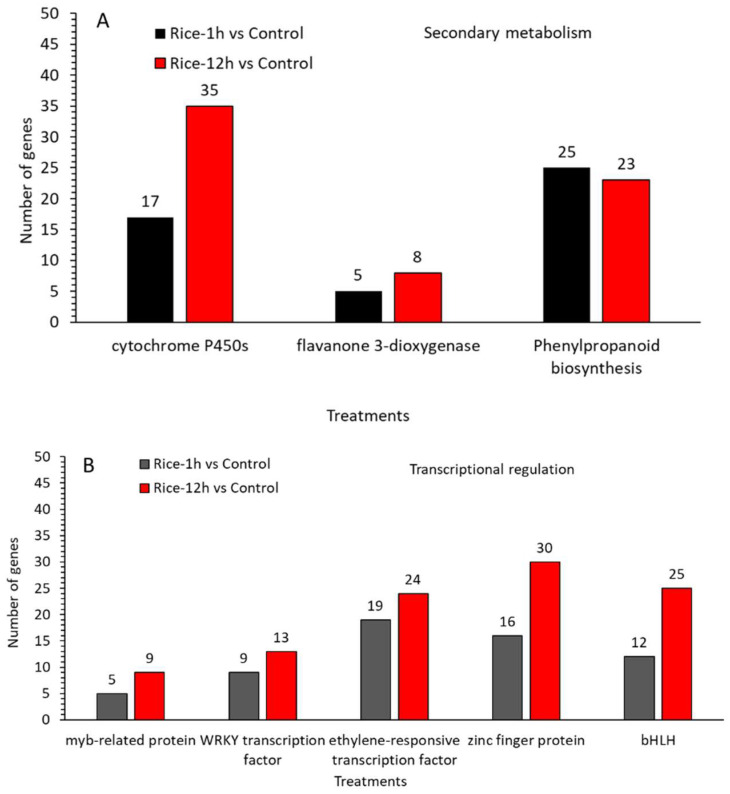
A number of upregulated genes related to secondary metabolism (**A**) and transcriptional regulation in Rice-1h vs. control (**A**) and Rice-12h vs. control (**B**) after FAW larvae infestation.

A further analysis indicated that MYB, WRKY, ethylene responsive factors (ERFs), zinc finger domain proteins, and basic helix–loop–helix (bHLH) are the TF families that are widely reported to be involved in plant defense responses. In this study, we identified MYB (5,9), WRKY (9,13), ethylene responsive factors (ERFs) (19,24), zinc finger domain proteins (16,30), and basic helix–loop–helix (bHLH) (12,25), which were upregulated in Rice-1h vs. control genes) and Rice-12h vs. control after FAW infestation. (Figure 7A,B) (Supplementary Table S3). Similarly, a chalcone synthase family protein is a key enzyme involved in the biosynthesis of flavonoids to encode *chalcone synthase* (*CHS*) and is required for the accumulation of purple anthocyanins in leaves and stems.

**Figure 8:**
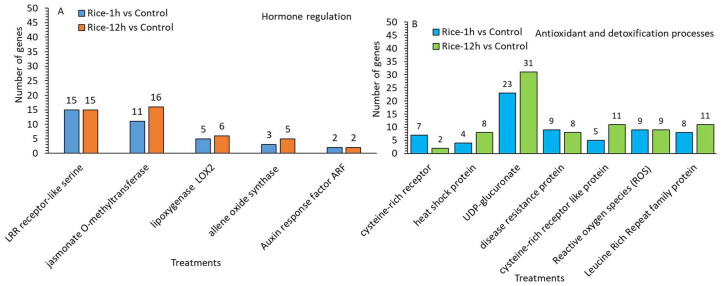
A number of upregulated genes related to hormone regulation (**A**) and antioxidant and detoxification processes in Rice-1h vs. control (**A**) and Rice-12h vs. control (**B**) after FAW larvae infestation.

In this transcriptome study, the results showed that 16 genes in Rice-1h vs. control and 23 in Rice-12h vs. control related to the metabolism of phenylpropanoid biosynthesis, which is central to producing defense-related compounds, were upregulated (Figure 8A,B). Similarly, further results indicated that five and eight genes related to the flavanone 3-dioxygenase were upregulated in Rice-12h vs. control and in Rice-12h vs. control respectively (Supplementary Table S3). Furthermore, key regulatory genes belonging to the subgroup of late anthocyanin biosynthesis genes were significantly upregulated in genes in Rice-1h vs. control (7 genes) and Rice-12h vs. control (11) after FAW infestation. Additionally, the involvement of Cytochrome P450 (CYP) genes was observed in Rice-1h vs. control (17 genes) and Rice-12h vs. control (53 genes) after FAW infestation. Furthermore, genes related to secondary metabolism, such as those involved in the tetracyclic triterpenoid biosynthetic process and DIMBOA UDP-glucosyltransferase BX8, exhibited higher transcript abundance in Rice-1h vs. control and Rice-12h vs. control following FAW infestation, specifically related to steroid and zeatin biosynthesis.

**Figure 9:**
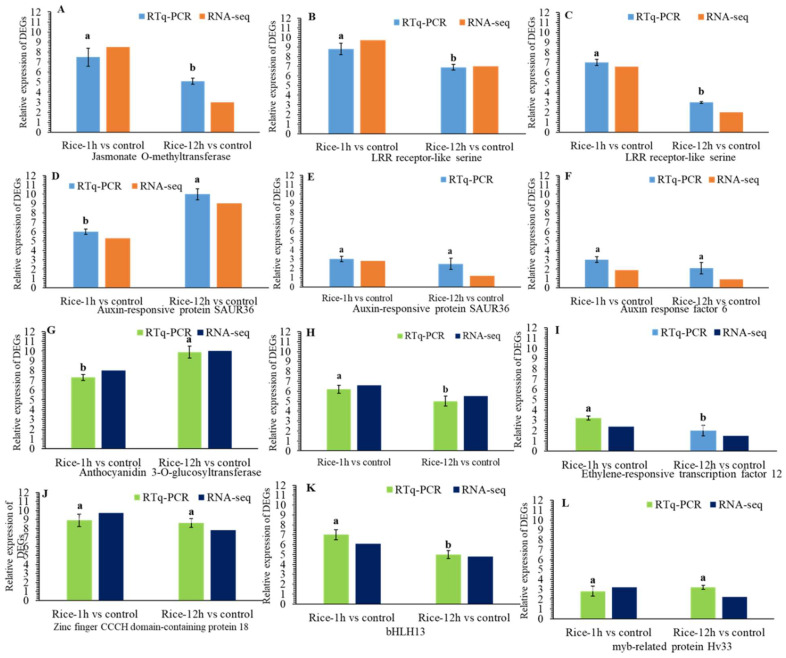
(**A**–**F**). Results for the qRT-PCR confirmation of the DEGs library. qRT-PCR analysis of ten upregulated genes of detoxification genes: transcriptional regulation (**A**–**F**) and hormone regulation (**G**–**L**) in Rice-1h vs. control and Rice-12h vs. control after FAW larvae infestation. Different letters a and b indicate significant differences.

In addition, lipoxygenase 2 LOX2 (5,6), a JA-signaling and -biogenesis gene (11,16), was detected as an upregulated gene in Rice-1h vs. control genes) and Rice-12h vs. control after FAW infestation (Figure 9 and Supplementary Table S4). Upregulation of allene oxide synthase (AOS) (3,5), is needed for JA production in Rice-1h vs. control genes and Rice-12h vs. control after FAW infestation. In addition, the upregulation expression of auxin-responsive protein related to signaling pathway, and Auxin response factor ARF (2,2), were upregulated in Rice-1h vs. control genes and Rice-12h vs. control after FAW infestation respectively. Similarly, we found disease resistance protein (9,8) which showed higher transcript abundance genes in Rice-1h and Rice-12h vs. control after FAW larvae infestation. Similarly, leucine-rich repeat (LRR) (15,15) protein kinase was highly expressed in Rice-1h vs. control genes and Rice-12h vs. control. In addition, genes encoding the cysteine-rich like kinase (7,2) and receptor-like proteins (RLPs) (5,11) were highly expressed upon FAW larvae feeding. Similarly, we identified the heat shock protein HSP 70 (4,8), which was present as an upregulated expression in Rice-1h vs. control genes and Rice-12h vs. control after FAW larvae feeding, highlighting the function of HSPs in plant defense against pathogenic infection and reduced accumulation of ROS. A further analysis indicated that, among detoxification genes, UDP-glycosyltransferase (23,31) significantly upregulated in Rice-1h vs. control genes and Rice-12h vs. control after FAW larvae feeding, suggesting this gene may play an important role in the rice resistance to FAW larvae feeding (Figure 8A,B and Supplementary Table S4). The results of the FPKM cluster analysis of differentially expressed genes related to secondary metabolism, transcriptional, hormones regulation, and antioxidant and detoxification processes in Rice-1h vs. control and Rice-12h vs. control after FAW larvae infestation were clustered using their log2 (FPKM + 1) value in the form of a heat map (Supplementary Figure S1A,B).

### 2.7. qRT-PCR for the Validation of Differentially Expressed Genes (DEGs)

**Figure 10:**
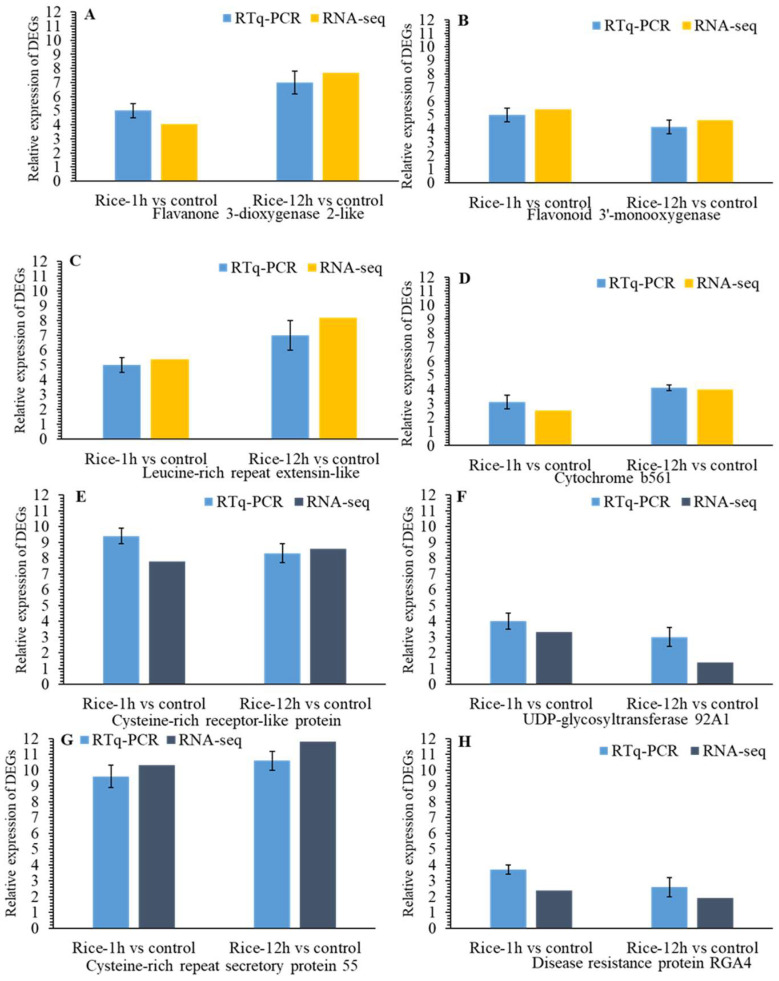
(**A**–**H**) Results for the qRT-PCR confirmation of the DEGs library and qRT-PCR analysis of upregulated genes of antioxidants and detoxification genes: Hormone regulation (**A**–**D**). Antioxidant and detoxification processes (**E**–**H**) in Rice-1h vs. control and Rice-12h vs. control after FAW larvae infestation.

We selected twenty differentially expressed genes (DEGs) for a quantitative reverse-transcription polymerase chain reaction (qRT-PCR) to verify the gene expression results obtained from the transcriptome data (Figure 9A–F). These selected genes include those that encode secondary metabolism, transcriptional regulation, hormone regulation, and antioxidant and detoxification processes (Figure 10A–H). In addition, our qRT-PCR results showed similar relative expression profiles of the selected DEGs with the RNA-Seq results in Rice-1h and Rice-12h vs. control after FAW larvae infestation.

## 3. Discussion

There are always potential risks to plants from a wide variety of herbivores. Plants have evolved defense mechanisms that are activated in response to insect herbivory to protect themselves against insects. Direct defenses, such as the activation of peroxidase, polyphenol oxidase, and proteinase inhibitor, are among these mechanisms [39,40]. Indirect defenses also function, such as the emission of a mixture of volatile fatty acid derivatives, phenylpropanoid chemicals, and terpenoids [41]. In this study, the transcriptomic analysis results revealed the differential expression of defense-related genes and pathways when rice plants were infested with FAW larvae for 1 h and 12 h. Our results are consistent with those of a previous study in which defense-related genes and pathways were significantly upregulated when plants were infested with herbivores [28,42,43]. Similarly, the results showed that genes related to the metabolism of phenylpropanoid biosynthesis, and flavanone 3-dioxygenase were significantly upregulated in rice after 1 h and 12 h of infestation, as compared to the control. Previous studies have reported the metabolism of phenylpropanoid, a central to producing defense-related compounds, including anthocyanins and flavonoids in plants [44,45,46]. Transcription factors, including MYBs, facilitate the activation of this critical regulatory gene, which is part of the late anthocyanin biosynthesis genes subgroup [47,48]. Additionally, the possible involvement of Cytochrome P450 (CYP) genes was found in Rice-1h vs. control and Rice-12h vs. control after FAW infestation. Consistent with our results, a previous investigation suggests that Cytochrome P450 (CYP) genes may play a role in plant defense [49,50]. Our results concluded that the plant immune system plays a crucial role in triggering induced defenses against herbivores.

Furthermore, the involvement of the TF families was found in our study in plant defense systems and has also been well-documented in previous studies [51]. MYB, WRKY, zinc finger domain proteins, ethylene response factors (ERFs), and basic helix–loop–helix (bHLH) are among these families [52]. Similarly, by encoding chalcone synthase (CHS), transparent testa4 (TT4), a member of the chalcone synthase family proteins, is a crucial enzyme in the synthesis of flavonoids [53]. It is important because it promotes the development of purple anthocyanins in stems and leaves [54,55]. Numerous studies have shown that the expression of genes linked to anthocyanin biosynthesis is regulated by a conserved regulatory complex called MYB-bHLH-WD40 (MBW) [56,57]. Additionally, after fall armyworm (FAW) infestation, we found a substantial upregulation of WRKY genes in rice plants. In previous studies, it has been reported that OsWRKY70 and JA are part of the same signaling cascade and are positive regulators of rice resistance to chewing herbivores [58,59]. The expression levels of zinc finger proteins of the Cys2/His2-type and C2H2-type were upregulated in rice when infested by FAW larvae. According to Han et al. [60], Cys2/His2-type zinc finger proteins improve resistance against insect pests, in addition to being involved in plant stress responses. Furthermore, after infestation by FAW larvae, the rice plants exhibited activation of ERFs, a critical subset of transcription factors involved in integrating ethylene (ET) and jasmonic acid (JA) signals [61,62,63]. In addition, previous studies have reported the roles of genes related to phytohormone signaling pathways, including jasmonic acid (JA), salicylic acid (SA), abscisic acid (ABA), and ethylene (ET), which result in the production of defensive compounds in plants in response to herbivores, including FAW [64,65,66]. In this experiment, transcript abundances of ERFs were increased in rice infested with FAW larvae as compared to the control. However, Arabidopsis has demonstrated the function of ERF1 as a regulator of ethylene responses following insect attack [67]. This indicates that rice plants may have developed specific defense mechanisms over time to counteract herbivorous insect attacks. These strategies could have evolved as an adaptive response to the pressures exerted by insect herbivory, allowing rice plants to enhance their survival and reproductive success in environments where insect pests pose a significant threat.

**Figure 11:**
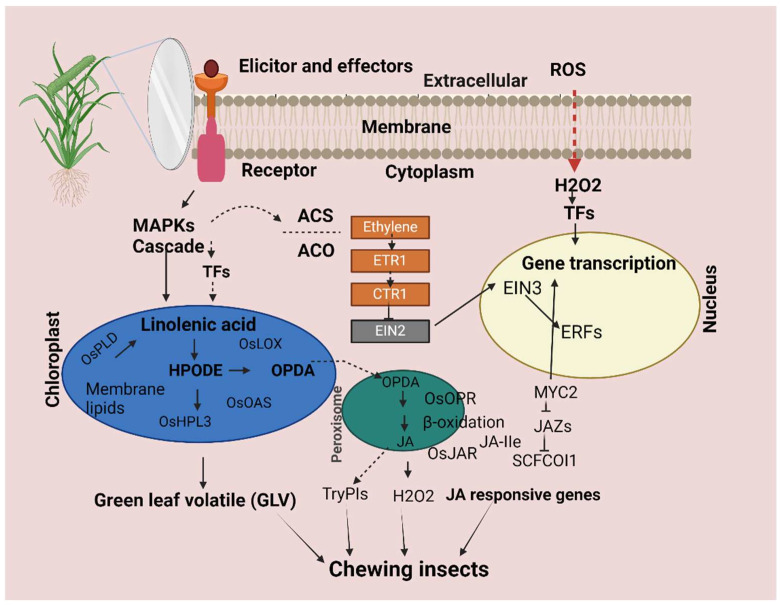
A working model diagram of molecular insights into the rice plant defense mechanism in response to an insect-attacked plant. Insect-derived elicitors are perceived by unidentified receptors on the plasma membranes, triggering rapid activation of MAPKs followed by biosynthesis of phytohormones, JA, JA-Ile, and ethylene. After several steps of signaling transduction, transcription factors (MYC2 and ERFs, for instance) regulate the accumulation of non-volatile secondary metabolites (such as TPIs in rice), which function as direct defenses against herbivores.

In this study, lipoxygenase 2 LOX2, a JA-signaling and -biogenesis genes, was detected as an upregulated gene in rice-1h vs. control genes and Rice-12h vs. control after FAW infestation. Previous studies showed that JA signaling has closely been associated with defense mechanisms against pathogens and insects [33,68,69]. A previous study reported that damage caused by fall armyworms in rice plants triggers a substantial early response linked to hormones such as jasmonates [30]. Similarly, upregulation of allene oxide synthase (AOS), needed for JA production, in rice plants after FAW infestation suggests that rice resistance to the insect attack is enhanced by the activation of JA signaling pathways [28]. Our results agree with the previous studies where the expression levels of LOX and AOS significantly increased after infestation [69,70]. In addition, the upregulation expression of auxin-responsive protein related to the signaling pathway, and auxin response factor ARF were upregulated in Rice after FAW larvae infestation. SAUR genes are related to cell division [71,72] and are reportedly regulated by the auxin level, indicating that this process could be impaired by FAW larvae feeding. In this experiment, we found disease-resistance protein which showed higher transcript abundance in rice after FAW larvae infestation. Similarly, leucine-rich repeat (LRR) protein kinase was highly expressed in rice after FAW larvae feeding, as compared to the control. A previous study showed that treatment with OS of *Spodoptera frugiperda* increased the expression level of *OsLRR-RLK1*, JA, SA, and ethylene biosynthesis genes and the activity of trypsin protease inhibitor (TrypPI) in rice [73]. In addition, genes encoding the Cysteine-rich like kinase and receptor-like proteins (RLPs) were highly expressed upon FAW larvae feeding. This highly upregulated CRK gene seems to indicate its potential role in resistance against insect pests [74]. We also observed receptor-like proteins (RLPs) and receptor-like kinase (RLK) genes that have a direct effect on the insect and pathogen [75,76]. Similarly, we identified the heat shock protein HSP 70 and detoxification genes like UDP-glycosyltransferase significantly upregulated in rice as compared to the control after FAW larvae feeding. Heat shock proteins (HSPs) and reactive oxygen species (ROS) are essential for promoting ROS scavenging activity and improving stress tolerance (Figure 11) [77,78]. Our study about the role of UDP-glycosyltransferase is further supported by earlier studies on spider mites in resistant and susceptible common bean cultivars [43]. In addition, our qRT-PCR results showed similar relative expression profiles of the selected DEGs with the RNA-Seq results in Rice-1h vs. control and Rice-12h vs. control after FAW larvae infestation. This study showed consistency with other study findings that show similar fold changes between qPCR and RNA-Seq data.

In conclusion, our study unveils the intricate defense mechanisms triggered by rice plants in response to an infestation of fall armyworm (FAW) larvae. Significant activation of defense-related genes and pathways was revealed by transcriptomic analysis in rice after FAW larvae infestation. Important transcription factors, including WRKY, MYB, and ERFs, as well as genes involved in the synthesis of flavonoids and phenylpropanoid, are crucial. Transcriptomic results showed that the plants’ defense mechanisms are further activated by the overexpression of auxin response genes and JA signaling pathways. The study is noteworthy for identifying particular genes, such as detoxification genes, RLPs, RLKs, and CRK, and highlighting their possible functions in increasing resistance against FAW larvae. Current research broadens our understanding of the intricate relationship between plants and herbivores, offering insights into the development of effective strategies in crop protection. Further research is needed to explore whether modulating these key genes impacts the performance of FAW larvae feeding on rice and to gain a deeper understanding of the efficiency of rice immunity against herbivores.

## 4. Materials and Methods

Larval populations of *S. frugiperda* were initially collected from two different corn fields located in Haikou, China. These larvae were subsequently placed alongside maize plants in a controlled environment with a temperature of 25 ± 2 °C and a 14:10 h light/ dark photoperiod. The plants were grown in a controlled plant growth chamber at 28 ± 2 °C with a 14:10 h light/dark photoperiod at the Ministry of Education Key Laboratory for Ecology of Tropical Islands, College of Life Sciences, Hainan Normal University, Haikou, China). The newly emerging moths were paired and placed in mating cages once they reached the pupation stage. There, they were fed a 10% honey solution. The population was used in host selection tests after undergoing a generation cycle on one-month-old maize seedlings for three generations before investigation.

### 4.1. Plants and Insect Infestation

The seeds of the commercial rice plant *Oryza sativa* “White Rice” (Báifàn) were purchased from China and planted in vermiculite mixed with peat moss (4:1) in plastic trays. The rice plants were grown in a controlled plant growth chamber at 28 ± 2 °C with a 14:10 h light/dark photoperiod at the Ministry of Education Key Laboratory for Ecology of Tropical Islands, College of Life Sciences, Hainan Normal University, Haikou, China. Thirty-day-old plants were used for experiments. After 3 generations were reared in the laboratory, a total of 90 third-instar larvae were released in plastic pots, 20 cm in height and 15 cm in width, containing 30-day-old rice plants. A total of three treatments with three replicates of each treatment contained 10 larvae for each replicate (each individual plant was treated as a single replicate). Plastic pots, 20 cm in height and 15 cm in width, containing 30-day-old rice plants without FAW larvae served as a control treatment. Damaged leaves of rice plants were collected after infestations with larvae after one hour and twelve hours (Figure 1). The treated leaves were then quickly frozen with liquid nitrogen and kept at −80 °C until the RNA isolation.

### 4.2. RNA Extraction, Library Preparation, and RNA-Sequencing Analysis

Total RNA was isolated (using the TRIzol1 Reagent (Invitrogen, Waltham, MA, USA) https://www.thermofisher.com/cn/en (accessed on 26 July 2023) by the manufacturer’s instructions) from three biological replicates, each made up of combined samples from at least three different plants. The RNA samples were then subjected to an RNase-free DNaseI treatment (Invitrogen; https://www.thermofisher.com) (accessed on 13 September 2022). Using a NanodropTM 2000 spectrophotometer, the quantity and quality of the RNAs were evaluated. All RNA samples were transferred to the Beijing Genomic Institute (BGI) in China for transcriptome sequencing and library preparation to guarantee proper analysis. Paired-end (2 × 150 bp) readings were produced using the Illumina HiSeq 2000 platform.

### 4.3. Reads’ Preprocessing and Differential Expression Analysis

The initial raw reads, obtained in Fastq format from the BGI institute website, underwent preprocessing and differential expression analysis. Trimmomatic v0.39 software [79] was utilized for quality control (QC), trimming low-quality reads, adapters, and Illumina-specific sequences. Quality thresholds of a minimum length of 50 bp and a minimum quality score of 30 were applied. Evaluation of raw sequence quality, both before and after filtering, was conducted using FastQC (https://www.bioinformatics.babraham.ac.uk/projects/fastqc/ (accessed on 26 July 2023)). Clean reads were aligned to the reference genome of rice using the RNA-Seq aligner STAR software version 2.7.11. Genes differential expression analysis was performed by DESeq2 software between two different groups (and by edgeR between two samples). The genes with the parameter of false discovery rate (FDR) below 0.05 and absolute fold change ≥2 were considered differentially expressed genes. Differentially expressed genes were then subjected to enrichment analysis of GO functions and KEGG pathways [80,81].

### 4.4. GO Terms and KEGG Pathways Enrichment Analysis

Through the use of the AgriGO module and Singular Enrichment Analysis (SEA), the roles of the DEGs were made clear. Finding enhanced Gene Ontology terms from the agriGO database [82] was the aim of this research, which had a significance threshold set at 0.05. Additionally, using the KOBAS 3.0 web server (http://kobas.cbi.pku.edu.cn/ (accessed on 26 July 2023)), an enrichment analysis of Kyoto Encyclopedia of Genes and Genomics (KEGG) pathways for the DEGs was conducted. KEGG pathways were deemed significantly enriched if their adjusted *p*-value was less than 0.05.

### 4.5. Validation of Transcripts DEGs Using qRT-PCR

For the validation of candidate DEGs, qRT-PCR was conducted for six selected DEGs, with Actin serving as the reference gene. Primers were designed using Geneious (Supplementary Table S5), and cDNAs were synthesized using the TaKaRa cDNA Synthesis Kit (TaKaRa, Dalian, China), following the manufacturer’s instructions. The qRT-PCR reactions were conducted on a Bio-Rad iQ5 Optical System (Bio-Rad Laboratories, Hercules, CA, USA). The 20 μL reaction solutions contained dreamtaq Master Mix 10 μL, 0.5 μL forward and 0.5 μL reverse primers, 1 μL (50 ng) of cDNA template, and 8 μL nuclease-free water. The qRT-PCR protocol involved an initial step at 95 °C for 3 min, followed by a denaturation step at 95 °C for 15 s, and an annealing/extension step at 60 °C for 30 s for a total of 40 cycles. Two reference genes and an Actin gene were used for data normalization. Subsequently, relative gene expression was calculated using the delta delta Ct method [83]. All data were analyzed using the SPSS Statistics v24.0 software (IBMSPSS Statistics Inc., Chicago, IL, USA) and all obtained results were reported as mean ± SE.

## Figures and Tables

**Table 1:** Summary of the sequencing data.

Sample	Raw Data		Valid Data		Valid Ratio (Reads)	Q20%	Q30%	GC Content%
	Read	Base	Read	Base				
Control1	52,435,330	7.87G	46,488,478	6.97G	88.66	99.96	97.85	49.5
Control2	49,733,028	7.46G	42,199,050	6.33G	84.85	99.96	97.96	49
Control3	48,440,398	7.27G	40,780,310	6.12G	84.19	99.95	97.94	49
Rice_12H1	39,611,302	5.94G	38,707,090	5.81G	97.72	99.97	96.39	50
Rice_12H2	36,895,594	5.53G	35,867,176	5.38G	97.21	99.97	96.16	49.5
Rice_12H3	47,139,810	7.07G	37,954,992	5.69G	80.52	99.96	97.89	50
Rice_1H1	44,931,270	6.74G	43,857,698	6.58G	97.61	99.97	96.94	52.5
Rice_1H2	33,816,536	5.07G	32,920,526	4.94G	97.35	99.97	96.24	50
Rice_1H3	37,937,056	5.69G	35,665,200	5.35G	94.01	99.97	96.45	48

## Data Availability

Data are contained within the article and Supplementary Materials.

## Supplementary Materials

The following supporting information can be downloaded at: https://www.mdpi.com/article/10.3390/plants13202879/s1, Figure S1: The results of the FPKM cluster analysis of differentially ex-pressed genes related to secondary metabolism, transcriptional, hormones regulation and antioxidant and detoxification processes were clustered using their log2 (FPKM + 1) value in the form of a heat map. Hierarchical clustering heatmap showing results of the FPKM clustering using log2 (FPKM + 1) values. The red and blue squares indicate genes with high or low gene expression levels in Rice-1h vs. control (A) and Rice-12h vs. control (B) after FAW larvae infestation, respectively; Table S1: Hormone regulation Rive-1h vs. control and Rive-12h vs. control; Table S2: Transcriptional regulation Rive-1h vs. control and Rive-12h vs. control; Table S3: Secondary metabolism Rive-1h vs. control and Rive-12h vs. control; Table S4: Antioxidant and detoxification processes Rive-1h vs. control and Rive-12h vs. control; Table S5: Primers used in this study for RTq-PCR.
