# Supplementary material for: Comprehensive Transcriptomic Analysis Reveals Defense-Related Genes and Pathways of Rice Plants in Response to Fall Armyworm (Spodoptera frugiperda) Infestation
Source: Plants (Basel). 2024 Oct 15;13(20):2879. doi: 10.3390/plants13202879 (PMC11510987; doi:10.3390/plants13202879)
Supplement: Supplementary file 1 [file plants-13-02879-s001.zip › Supplementary Figure S1.pdf]

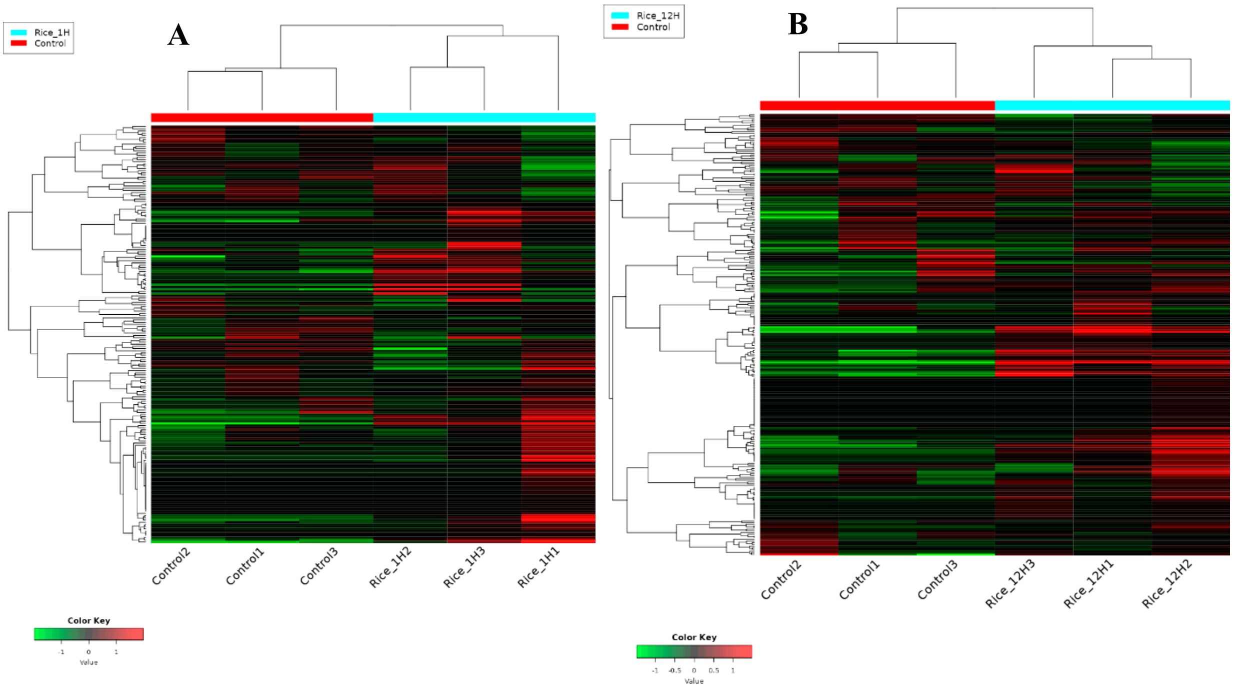

Figure S1. The results of the FPKM cluster analysis of differentially ex-pressed genes related to secondary metabolism, transcriptional, hormones regulation and antioxidant and detoxification processes were clustered using their log2 (FPKM+1) value in the form of a heat map. Hierarchical clustering heatmap showing results of the FPKM clustering using log2 (FPKM+1) values. The red and blue squares indicate genes with high or low gene expression levels in Rice-1h vs control (A) and Rice-12h vs control (B) after FAW larvae infestation, respectively.
