# Supplementary material for: Comprehensive Transcriptomic Analysis Reveals Defense-Related Genes and Pathways of Rice Plants in Response to Fall Armyworm (Spodoptera frugiperda) Infestation
Source: Plants (Basel). 2024 Oct 15;13(20):2879. doi: 10.3390/plants13202879 (PMC11510987; doi:10.3390/plants13202879)
Supplement: Supplementary file 1 [file plants-13-02879-s001.zip › Table S5.pdf]

Table S5. Primers used in this study for RTq-PCR

Transcripts name	Sense primers	Anti-Sense primers	Product size (pb)
Jasmonate O-methyltransferase	ATGGCATCCTGAGAAGCTGAG	AGGTCCGTAAATCGGCATGT	94
LRR receptor-like serine	TCTTCGCAACAAGGATCCTGAA	TGCACAGAGTACTCCTCCGA	108
LRR receptor-like serine	TGTGTACAGCCTTGGGGTTG	ACTCAAAGTTGAAGAGCGTGT	105
auxin-responsive protein SAUR36	ACTCTGAGTTTCTGACATGCGA	ACCCTGAAGGCGACTATTGC	92
auxin-responsive protein SAUR36	ACTTCCTTGTGAGGCAGCAG	GACAGAGCTCACAACAGCCT	94
Auxin response factor 6	ACATCCGTGAGGTTCGGAAT	CTCCTGCGGCGAAAGTATCT	75
Anthocyanidin 3-O-glucosyltransferase	TTCCTCGCAGTGCTCAACTT	AGGATCCCTCTCCCCTTCAC	104
WRKY transcription factor 30	GATTAGGGTGAGCTCGACGG	GGTAGTAACCCCTTGGGTGC	116
Ethylene-responsive transcription factor 12	TAGCGTTCTCACATTGCCCC	ACAGAAGAGATGAAAAGCTGTACG	75
Zinc finger CCCH domain-containing protein 18	TGGCCCATCCATTTTGCTCA	CGTTCGAGCTCAGATCAAATGT	113
bHLH13	GCCTGAAGCATCGTATAGGGG	CTTAACACGCCTTGAGCGAA	97
myb-related protein Hv33	TAGCCAGTGAGGAGAAGGGT	GTAGCTACCACCACCACCAC	115
Flavanone 3-dioxygenase 2-like	GAGATGCACTGTCCTGAGGT	GTCCTATGCACCATGACCGT	73
Flavonoid 3'-monooxygenase	GCTCAACGAGATCGTGCTCA	GAACACCGTCACCTTCCTGA	85
Leucine-rich repeat extensin-lik	AGGTCGTCGTTAAGCCCAAG	TCAATGGCCCACGTACTGAC	73
Cytochrome b561	AACGAGCCGTTGGAATGAGT	AGCAAAACGACGGAACTCCA	78
Cysteine-rich receptor-like protein	ACCTGGTGTACAAGCTGACG	GTAGAGCCTGAGCGATGACC	77
UDP-glycosyltransferase 92A1	TAACGCACGCATTTGGGTTT	CGCGGACAAGGCTTTACCAT	99
Cysteine-rich repeat secretory protein 55	ACACACGTTTGTGTGGAGCA	GCCTTCCAAGCTGGTCCTA	102
Disease resistance protein RGA4	AAGCAAACTCAAAGCAGGCA	GAATTATTCCTGTGGCTGCCG	125
actin	GCTTGCATACGTCGCTCTTG	GCTTCCATGCCGATGAGAGA	
